# Numerical Modeling of Transient Two-Phase Flow and the Coalescence and Breakup of Bubbles in a Continuous Casting Mold

**DOI:** 10.3390/ma15082810

**Published:** 2022-04-12

**Authors:** Yushi Tian, Pengzhao Shi, Lijun Xu, Shengtao Qiu, Rong Zhu

**Affiliations:** 1 School of Metallurgical and Ecological Engineering, University of Science and Technology Beijing, Beijing 100083, China; yushi_tian@tom.com (Y.T.); zhurong@ustb.edu.cn (R.Z.); 2 National Engineering Research Center of Continuous Casting Technology, Central Iron and Steel Research Institute, Beijing 100081, China; shipengzhao1990@163.com (P.S.); qiust@vip.sina.com (S.Q.)

**Keywords:** bubbles, spatial distribution, coalescence, bounce, breakup, continuous casting mold, large eddy simulation

## Abstract

The multiphase flow and spatial distribution of bubbles inside a continuous casting (CC) mold is a popular research issue due to its direct impact on the quality of the CC slab. The behavior of bubbles in the mold, and how they coalesce and break apart, have an important influence on the flow pattern and entrapment of bubbles. However, due to the limitations of experiments and measurement methods, it is impossible to directly observe the multiphase flow and bubble distribution during the CC process. Thus, a three-dimensional mathematical model which combined the large eddy simulation (LES) turbulent model, VOF multiphase model, and discrete phase model (DPM) was developed to study the transient two-phase flow and spatial distribution of bubbles in a continuous casting mold. The interaction between the liquid and bubbles and the coalescence, bounce, and breakup of bubbles were considered. The measured meniscus speed and bubble diameter were in good agreement with the measured results. The meniscus speed increased first and then decreased from the nozzle to the narrow face, with a maximum value of 0.07 m/s, and appeared at 1/4 the width of the mold. The current mathematical model successfully predicted the transient asymmetric two-phase flow and completely reproduced the coalescence, bounce, and breakup of bubbles in the mold. The breakup mainly occurred near the bottom of the submerged entry nozzle (SEN) due to the strong turbulent motion of the molten steel after hitting the bottom of the SEN. The average bubble diameter was about 0.6 mm near the nozzle and gradually decreased to 0.05 mm from the nozzle to the narrow face. The larger bubbles floated up near the SEN due to the effect of their greater buoyancy, while the small bubbles were distributed discretely in the entire mold with the action of the molten steel jet. Overall, the bubbles were distributed in a fan shape. The largest concentration of bubbles was in the lower part of the SEN and the upper edge of the SEN outlet.

## 1. Introduction

The argon injection method is widely used in the continuous casting (CC) process to prevent the clogging of the submerged entry nozzle (SEN) [1]. The injected discrete argon bubbles can effectively prevent the clogging of the SEN, but it is also a key factor for quality defects in steel products. The argon bubbles either move to the top slag layer to be removed from the molten steel, or they move to the solidification front to be captured as defects [2]. Therefore, the prediction of the motion and spatial distribution of bubbles in the mold is of great significance to improve the quality of CC slabs. Many scholars have carried out measurement studies on the distribution of bubbles in the SEN and mold [3,4,5,6,7], including the effect of the gas flow rate, the diameter of the injection port, the density of the gas and liquid, and the surface tension. Cho [8] and Bai [3] studied the formation, expansion and growth, and breakup of bubbles under different injection methods using a mold water model. The above studies were carried out based on physical simulation, which has a definite gap with the distribution of bubbles under high temperature and high pressure during the actual CC process. The numerical simulation method can overcome these shortcomings, and it has therefore been widely developed and applied [9].

Yang [10] gave a detailed overview of the multiphase flow model applied in the CC process. The solution methods of multiphase flow are mainly divided into the Eulerian–Lagrangian model [11,12,13] and the Eulerian-Eulerian model [14,15,16]. Both the molten steel and argon bubbles were considered as a continuum phase, and the interaction between different phases was solved by the interphase force in the Eulerian-Eulerian model. Liu [17] used a Eulerian multiphase model to predict the time-dependent argon–steel–slag–air quasi-four-phase flow inside a slab CC mold. Three typical flow patterns inside the mold with different gas flow rates were proposed. Thomas [18,19] developed a coupled standard *k*-*ε* turbulence model and the Eulerian-Eulerian model to investigate the effect of argon gas bubble injection on flow-related phenomena in a typical steel slab caster. Àvila-Ortiz [20] developed a mathematical model to simulate the influence of the flow rate of the gas on the flow patterns of the liquid in a water–air model. Previous studies have mostly used the Eulerian-Eulerian model to simulate the multiphase flow in the mold, but the Eulerian-Eulerian model cannot provide individual information, such as the spatial distribution, velocity, and size of each bubble. The molten steel was treated as a continuum phase, and the argon bubbles were treated as a discrete phase in the Eulerian–Lagrangian model. The bubbles were tracked by solving their motion equations [21]. Using this model, Lei [22] developed a three-dimensional model to study the dynamic evolution of the gas–liquid interface and the solidification process. Chen [23,24,25] combined the large eddy simulation (LES) turbulent model, the VOF multiphase model, and the discrete phase model (DPM) to evaluate the effects of different interphase forces on the fluid flow and the spatial distribution of the bubbles in a CC strand. The bubble diameter was obtained by transforming the water model measurements through empirical formulas [26]. Wang [27] investigated the influence of the argon flow rate on the multiphase flow, heat transfer, and initial solidification in a CC slab mold. Although numerous results have been published, the vast majority of studies have ignored the interaction between bubbles, such as the coalescence and breakup of bubbles.

Many fundamental theories of bubble coalescence [28,29] and breakup [30,31] have been published. However, the behavior of the bubbles under the two-phase flow is such a complex phenomenon that there was and is no complete model applicable to all situations. In particular, the coalescence and breakup of bubbles in the continuous casting process of molten steel still needs further research. Based on the Eulerian-Eulerian model, Liu [32] used the *k*-*ε* model and the Multiple-Size-Group (MUSIG) model to predict the polydispersed bubble flow and bubble size distribution inside the slab CC mold. Santos [33] also employed the MUSIG model to discuss the effect of gas distribution on the flow field of liquid inside the mold and other metallurgical aspects. A relatively small number of studies using the Eulerian–Lagrangian model method to study the bubble diameter distribution have also been published. Zhang et al. [34,35] developed a new mathematical model considering the process of bubble interaction to simulate the fluid flow, dispersed bubble motion, and transport process in the slab CC mold. Using the modified bubble coalescence and breakup models, Yang [36,37] simulated bubble behavior in a slab CC mold. It found that the bubble size and number at both the narrow and wide faces decreased with the increase in distance from the meniscus. The effect of the initial bubble size on the distribution of bubbles captured by the solidification shell was also studied [38]. The above researchers have developed an understanding of the size distribution of bubbles in the mold to a certain extent. However, the effect of the transient asymmetric flow field in the CC mold on the bubble behavior was not considered. The transient asymmetric flow existing in the mold was indeed found to have a significant effect on the bubble distribution. [39,40]. Therefore, our understanding of the interaction between the molten steel and the bubbles, and the interaction between the bubbles on the basis of the transient fluid flow, still need further study.

Therefore, in the current study, a three-dimensional mathematical model combining the LES turbulent model, VOF multiphase model, and DPM was established to study the two-phase flow and spatial distribution of bubbles in a CC mold. The transient asymmetric fluid flow and the coalescence, bounce, and breakup of bubbles were included to achieve an accurate prediction of the spatial distribution of bubbles.

## 2. Mathematical Formulation

### 2.1. Governing Equation

#### 2.1.1. Turbulence Model

The transient turbulent fluid flow inside the SEN and the mold are resolved using the LES model. The governing equation of the momentum is defined as:

(1) $$\frac{\partial }{\partial t}\left(\rho u_{i}\right)+\frac{\partial }{\partial x_{j}}\left(\rho u_{i}u_{j}\right)=-\frac{\partial p}{\partial x_{i}}+\frac{\partial }{\partial x_{j}}\left[\left(\mu +\mu_{t}\right)\left(\frac{\partial u_{i}}{\partial x_{j}}+\frac{\partial u_{j}}{\partial x_{i}}\right)\right]+F_{mom,i}$$

where *ρ* is the density in kg/m^3^, *t* is the time in s; *u* is the velocity in m/s, *p* is the pressure in Pa, *μ* is the viscosity in kg/(m·s), and *F_mom,i_* is the source term induced by the interaction between bubbles and molten steel in kg/(m^2^·s^2^). The turbulent viscosity *μ_t_* is calculated using the Smagorinsky–Lilly model [41], as shown in Equation (2).

(2) $$\mu_{t}=\rho L_{s}^{2}\sqrt{2{\bar{S}}_{ij}{\bar{S}}_{ij}}=\rho {\left[\min\left(\kappa d,C_{S}V^{\frac{1}{3}}\right)\right]}^{2}\sqrt{2{\bar{S}}_{ij}{\bar{S}}_{ij}}$$

where *κ* is the Kármán constant; *d* is the distance to the closest wall in m; *C_S_* is the Smagorinsky constant, and *V* is the volume of the computational cell in m^3^. *L_S_* is the mixing length for sub grid scales in m, and *S* is the rate-of-strain tensor for the resolved scale in s^−1^.

#### 2.1.2. Multiphase Model

The liquid water and air layer at the top of the mold are considered as a continuum phase. The interface between the water and air is tracked using the VOF multiphase model by the solution of a continuity equation for the volume fraction of each phase. For the phase *q*, the continuity equation is calculated as:

(3) $$\frac{\partial }{\partial t}\left(\alpha_{q}\rho_{q}\right)+\nabla \cdot \left(\alpha_{q}\rho_{q}u_{q}\right)=0$$

where *α_q_* is the volume fraction of phase *q*; *u_q_* is the velocity of phase *q* in m/s.

#### 2.1.3. Bubble Tracking Model

The bubbles inside the mold are treated as a discrete phase, and their motions are governed by integrating the force balance on the particle in a Lagrangian reference frame:

(4) $$\frac{du_{b}}{dt}=F_{b}+F_{D}+F_{L}+F_{P}+F_{VM}$$

where the terms on the right side of the equation are gravity buoyancy force, drag force, lift force, pressure gradient force, and virtual mass force, respectively. The two-way coupled DPM is achieved by including those interphase forces between the water and bubbles. The effect of the lift force is calculated using a User-Defined Function (UDF).

The gravity buoyancy force is given by:

(5) $$F_{b}=\frac{\rho_{b}-\rho_{l}}{\rho_{b}}g$$

where the *ρ_b_* is the bubble density in kg·m^−3^, and *ρ_l_* is the water density in kg·m^−3^.

The drag force is given by:

(6) FD=34μCDReρbd2ul−ub

where *C_D_* is the drag coefficient and calculated in Equation (7), *μ* is the viscosity of the liquid in kg·m^−1^·s^−1^, *d* is the bubble diameter in m, Re is the relative Reynolds number, *u_l_* is the liquid velocity in m·s^−1^, and *u_b_* is the bubble velocity in m·s^−1^.

(7) CDvis=24Re×1+0.1×Re0.75CDvis>CDdis,CD=CDvisCDdis=23×gρl0.5dσ0.5×1+17.671−αg1.28618.671−αg1.5CDvis<CDdis<CDcap,CD=CDdisCDcap=831−ag2CDdis>CDcap,CD=CDcap

The lift force is given by:

(8) $$F_{L}=C_{L}\frac{\rho_{l}}{\rho_{b}}\left(u_{l}-u_{b}\right)\times \nabla \times u_{l}$$

where *C_L_* is the lift force coefficient and is calculated using the Tomiyama model [42] as follows:

(9) CL=Min[0.288tanh(0.121Re,f(Eo′))]if Eo′≤4f(Eo′)if 4<Eo′≤10−0.27if 10≤Eo′,

(10) $$f(Eo^{\prime })=0.00105{\left(Eo^{\prime }\right)}^{3}-0.0159{\left(Eo^{\prime }\right)}^{2}-0.0204Eo^{\prime }+0.47$$

(11) $$Eo=\frac{g\left(\rho_{l}-\rho_{b}\right)d^{2}}{\sigma }$$

(12) $$Eo^{\prime }=\frac{g\left(\rho_{l}-\rho_{g}\right){\left(1+0.163Eo^{0.757}\right)}^{2/3}d^{2}}{\sigma }$$

The pressure gradient force is given by:

(13) $$F_{P}=\frac{\rho_{l}}{\rho_{b}}u_{b}\nabla u_{l}$$

The virtual mass force is given by:

(14) $$F_{VM}=C_{vm}\frac{\rho_{l}}{\rho_{b}}\left(u_{b}\nabla u_{l}-\frac{du_{b}}{dt}\right)$$

where *C_vm_* is the virtual mass force coefficient and set as 0.5.

#### 2.1.4. Coalescence, Bounce, and Breakup Model

The O’Rourke algorithm [43] is used to calculate the probability of the collision of two bubbles. When the bubble *i* center passes within a flat circle centered around the bubble *j* of area *π*(*r_i_* + *r_j_*)^2^ perpendicular to the trajectory of the bubble *i*, a collision takes place. A collision volume to calculate the probability of collision in the O’Rourke algorithm is used, as shown in Equation (15).

(15) $$V_{c}=\pi {\left(r_{i}+r_{j}\right)}^{2}u_{R}\Delta t$$

where *r_i_* and *r_j_* are the radii of bubble *i* and bubble *j*, respectively; *u_R_* is the relative velocity between the water and bubbles.

This assumes that there is a uniform probability of the bubble being anywhere within the cell, so the chance of the bubble being within the collision volume is the ratio of the two volumes. Thus, the probability of the bubble *j* colliding with bubble *i* is calculated as:

(16) $$P=\frac{\pi {\left(r_{i}+r_{j}\right)}^{2}u_{R}\Delta t}{V}$$

where *V* is the mesh volume where the bubble is located.

The outcome of the collision tends to be coalescence if the bubbles collide head-on, and bouncing if the collision is more oblique. The critical offset *b_crit_* and the actual collision parameter *b* are proposed to determine the outcome of the collision [43]:

(17) $$b=\left(r_{i}+r_{j}\right)\sqrt{Y}$$

(18) $$b_{crit}=\left(r_{i}+r_{j}\right)\sqrt{\min\left(1.0,\frac{2.4f}{We}\right)}$$

(19) $$\begin{array}{l} f={\left(\frac{r_{i}}{r_{j}}\right)}^{3}-2.4{\left(\frac{r_{i}}{r_{j}}\right)}^{2}+2.7\left(\frac{r_{i}}{r_{j}}\right)\\ We=\frac{\rho u_{R}^{2}\sqrt{d_{i}d_{j}}}{\sigma } \end{array}$$

where *Y* is a random number between 0 and 1.

Coalescence occurs when the critical offset *b_crit_* is larger than the actual collision parameter *b*. The velocity and radius of the generated bubble are calculated in Equation (20). Otherwise, the new velocities are calculated in Equation (21) for the case of a grazing collision.

(20) $$u_{i}^{\prime }=\frac{m_{i}u_{i}+m_{j}u_{j}}{m_{i}+m_{j}},r_{i}^{\prime }={\left(\frac{3\left(m_{i}+m_{j}\right)}{4\pi \rho_{b}}\right)}^{\frac{1}{3}}$$

(21) $$\begin{array}{l} u_{i}^{\prime }=\frac{m_{i}u_{i}+m_{j}u_{j}}{m_{i}+m_{j}}+\frac{m_{j}u_{R}}{m_{i}+m_{j}}\left(\frac{b-b_{crit}}{r_{i}+r_{j}-b_{crit}}\right)\\ u_{j}^{\prime }=\frac{m_{i}u_{i}+m_{j}u_{j}}{m_{i}+m_{j}}+\frac{m_{i}u_{R}}{m_{i}+m_{j}}\left(\frac{b-b_{crit}}{r_{i}+r_{j}-b_{crit}}\right) \end{array}$$

where *m_i_* and *m_j_* are the masses of bubble *i* and bubble *j*, respectively; *u_i_* and *u_j_* are the velocities of bubble *i* and bubble *j*, respectively;

The strong turbulent flow in the SEN and the mold causes the breakup of bubbles. Therefore, there is a critical diameter for the stable existence of bubbles under different turbulent kinetic energies. Evans [44] proposes a maximum stable bubble size *d_crit_* as:

(22) $$d_{crit}=\left(\frac{We_{c}\sigma }{2\rho_{l}}\right)\varepsilon^{-0.4}$$

where *We_c_* is the critical Weber number and set as 1.2; *ε* is the turbulent energy dissipation rate in m^2^/s^3^.

Breakup happens when the bubble diameter *d_p_* is larger than *d_crit_*. Then, the smaller daughter bubble size *d*_1_ is randomly determined in the range of *d_min_* and *d_max_* determined by force balance criteria and mass balance criteria [45,46].

(23) $$d_{min}=\frac{\sigma }{\rho_{l}\frac{u_{\lambda }^{2}}{2}}\;d_{max}=\frac{1}{\sqrt[{3}]{2}}d_{p}$$

where *u_λ_* is the eddy velocity in m/s. Once *d*_1_ is determined, another daughter bubble diameter *d*_2_ is calculated by Equation (24).

(24) $$d_{2}={\left(d_{p}^{3}-d_{1}^{3}\right)}^{\frac{1}{3}}$$

### 2.2. Computational Domain and Computational Details

**Figure 1:**
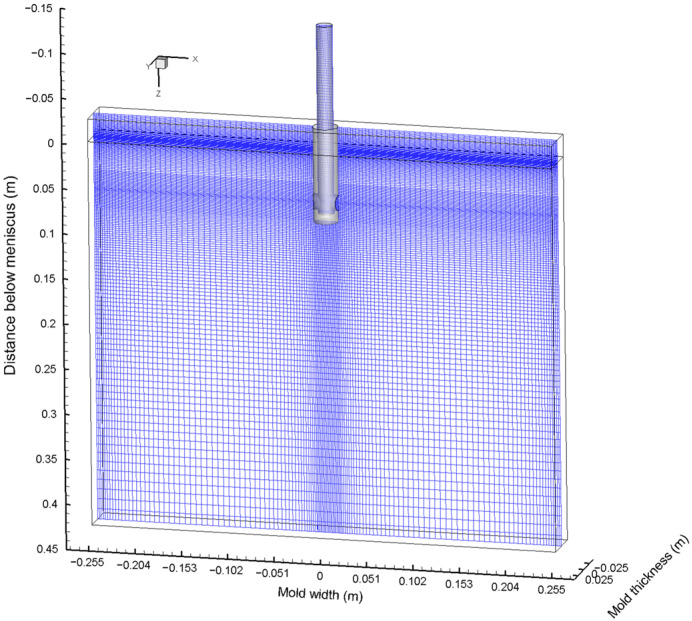
Schematic diagram of the computational domain and mesh system.

In the current study, a three-dimensional mathematical model based on a 1/4 scale water model of a slab CC mold was carried out. Figure 1 shows the distribution of the computational domain and mesh system. The mesh near the water–air interface was refined to accurately capture the distribution of the liquid level. The total number of meshes was about 440,000. The section size of the CC mold was 510 mm × 50 mm, and the thickness of the air layer at the top of the CC mold was 25 mm. The casting speed was 0.425 m/min. The air gas was injected at the inlet of the SEN, and the flow rate was 90 mL/min. More detailed model parameters and physical properties can be found elsewhere [23]. The three-dimensional transient two-phase flow in the SEN and the CC mold was simulated using the LES model and VOF model. The trajectories of gas bubbles were tracked using the DPM. The interaction between the liquid and bubbles and the coalescence, bounce, and breakup of bubbles were included using a UDF.

### 2.3. Boundary Conditions

The constant velocity inlet boundary condition at the SEN inlet was used. The free-slip condition was used at the top of the mold, and the pressure outlet boundary condition was used at the outlet. The non-slip condition was adopted for other walls. The bubbles with an initial diameter of 0.71 mm (the measured average diameter of the water model) were injected at the inlet of the SEN. The bubbles were assumed to be removed when the volume fraction of the air was large than 0.5 and escaped at the outlet of the mold. The reflection condition was used for other walls.

## 3. Validation

**Figure 2:**
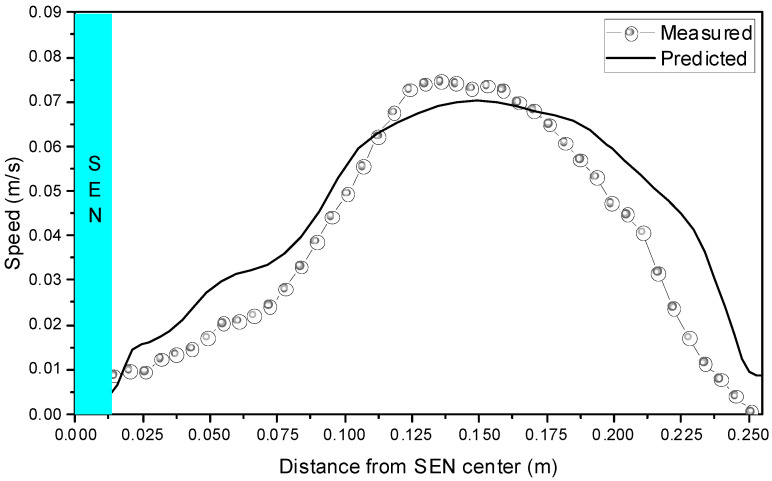
Comparison between the measured and the predicted result of the speed on the thickness centerline at 5 mm below the meniscus.

The measured speed on the thickness centerline at 5 mm below the meniscus using Particle Image Velocimetry (PIV) was employed to validate the current mathematical model, as shown in Figure 2. The PIV is widely used to measure the velocity in water models. First, a certain number of small particles were added to the mold during the measurement. Then, a 532 nm laser was used to illuminate the measured flow field area, and the movement of the particles through multiple exposures was recorded. Finally, the particles were tracked, and the velocity distribution was calculated. The average diameter of the small particles was 15 μm, and the density of the particles was about 1.0. The predicted results were in good agreement with the measured results. The speed increased first and then decreased from the SEN to the narrow face. The maximum speed appeared near the 1/4 width of the mold.

**Figure 3:**
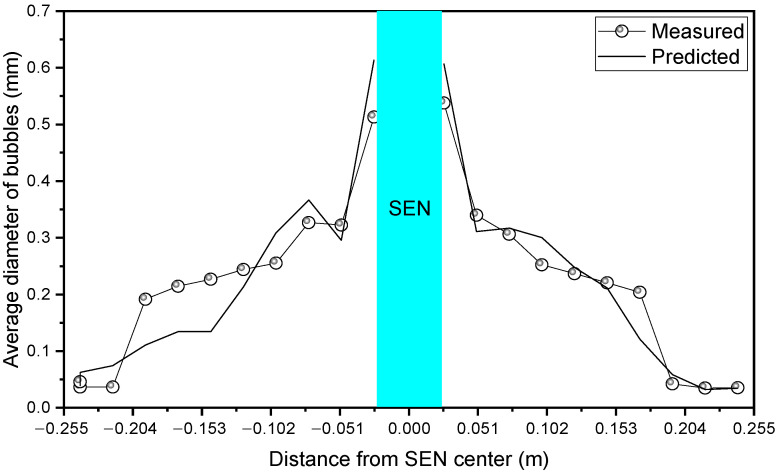
Comparison between the measured and the predicted result of the average bubble diameter along the width of the mold.

The space from the SEN to the narrow face was divided into 10 equal zones to count the distribution of bubbles. Figure 3 compares the measured and predicted distribution of the average diameter of the bubbles along the width of the mold. The difference between the predicted result and the measured result was small. The average bubble diameter was larger near the SEN, and the average bubble diameter decreased gradually from the SEN to the narrow face. The above results show that the current coupled LES turbulent model, VOF multiphase model, and DPM can accurately predict the multiphase flow and spatial distribution of the bubbles in the mold.

## 4. Results and Discussion

### 4.1. Distribution of the Transient Two-Phase Flow Field

**Figure 4:**
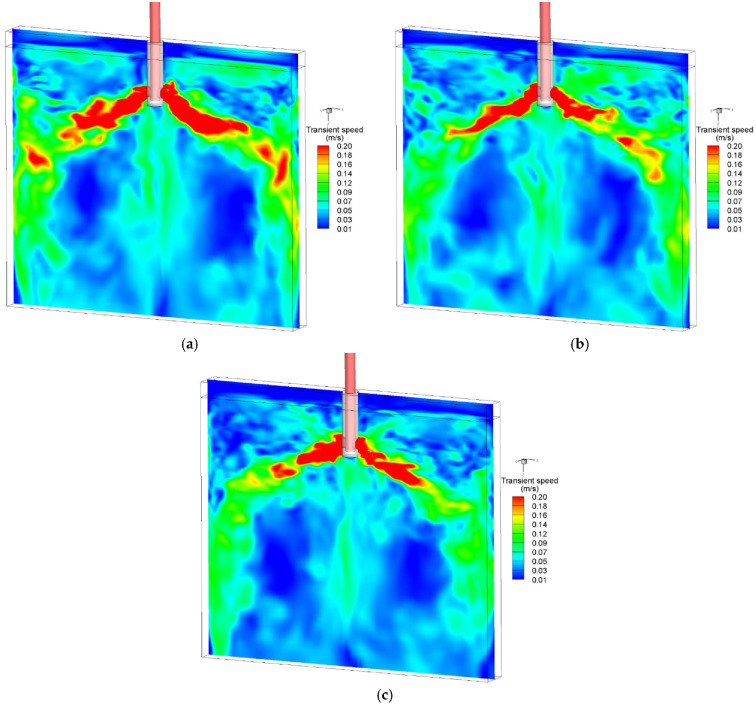
Distribution of the transient velocity at three different time shots: (**a**) t = 40.0 s; (**b**) t = 45.0 s; (**c**) t = 50.0 s.

The distribution of bubbles in the mold is mainly determined by the movement of the molten steel. Therefore, the correct simulation of the flow field is a prerequisite for predicting the distribution of the bubble size, number, and diameter. Figure 4 shows the distribution of the transient two-phase flow at different times. The current LES model successfully predicted the transient asymmetric two-phase flow in the mold. At t = 45 s, the speed of the jet on the left side of the SEN was significantly higher than that on the right side. At t = 40 s and 50 s, the speed of the jet on both sides of the SEN was relatively consistent. However, the upper circulation speed at t = 40 s was significantly smaller than that at t = 50 s. When the casting speed and the gas flow rate were 0.425 m/min and 90 mL/min, respectively, the flow pattern in the mold was a typical double-roll flow.

**Figure 5:**
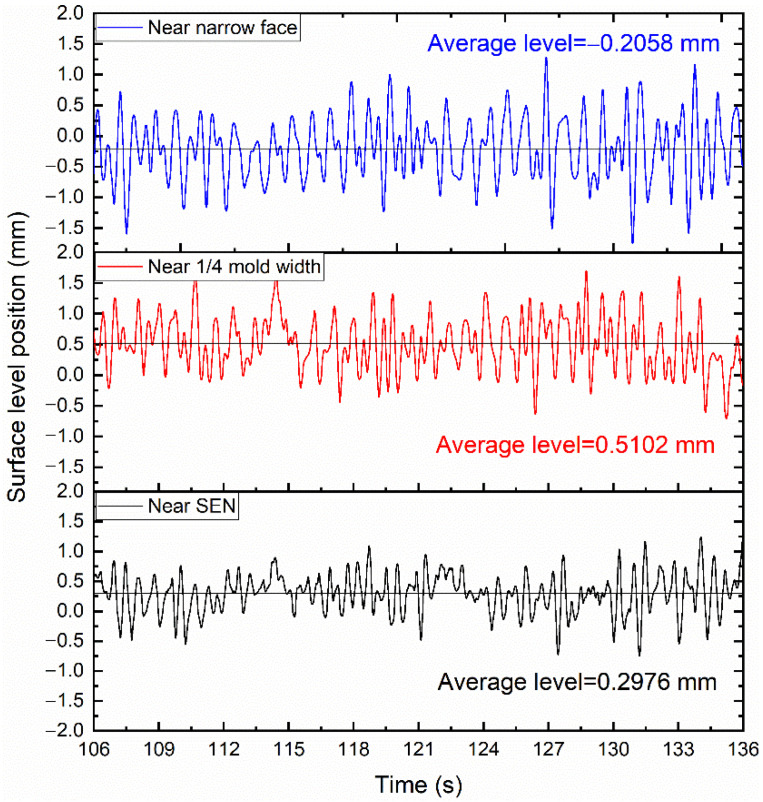
The variation in surface level over time at different locations.

Figure 5 shows the variation in the surface level over time near the narrow face, 1/4 width of the mold, and the SEN. The results show that the surface level near the narrow surface was the highest, and the surface level was the lowest near the 1/4 width of the mold. This is an inevitable result of the double-roll flow. The surface level near the narrow face was raised due to the lifting of the upper recirculation flow.

### 4.2. Spatial Distribution of the Bubbles

**Figure 6:**
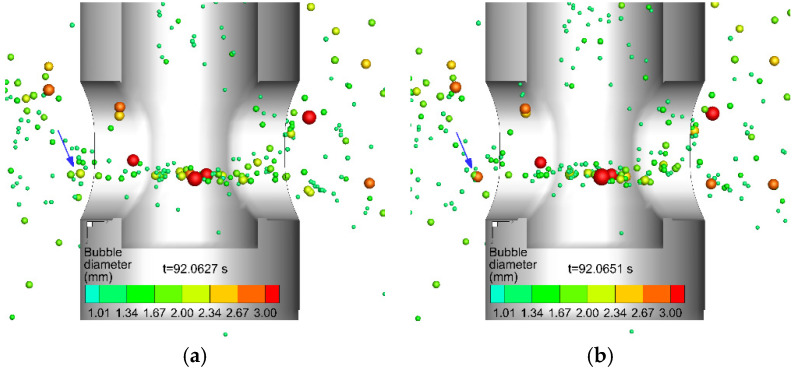

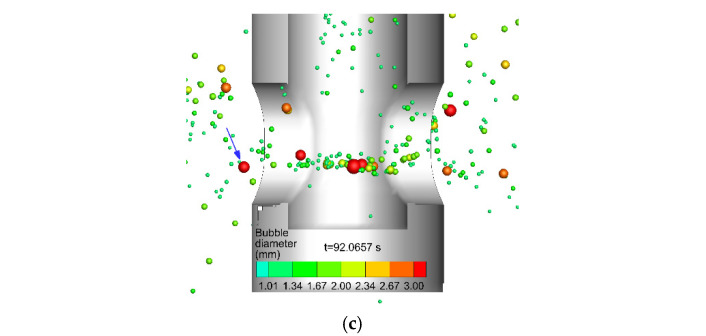
The typical process of the coalescence of bubbles (marked by a blue arrow), (**a**) t = 92.0627 s, (**b**) t = 92.0651 s, and (**c**) t = 92.0657 s.

**Figure 7:**
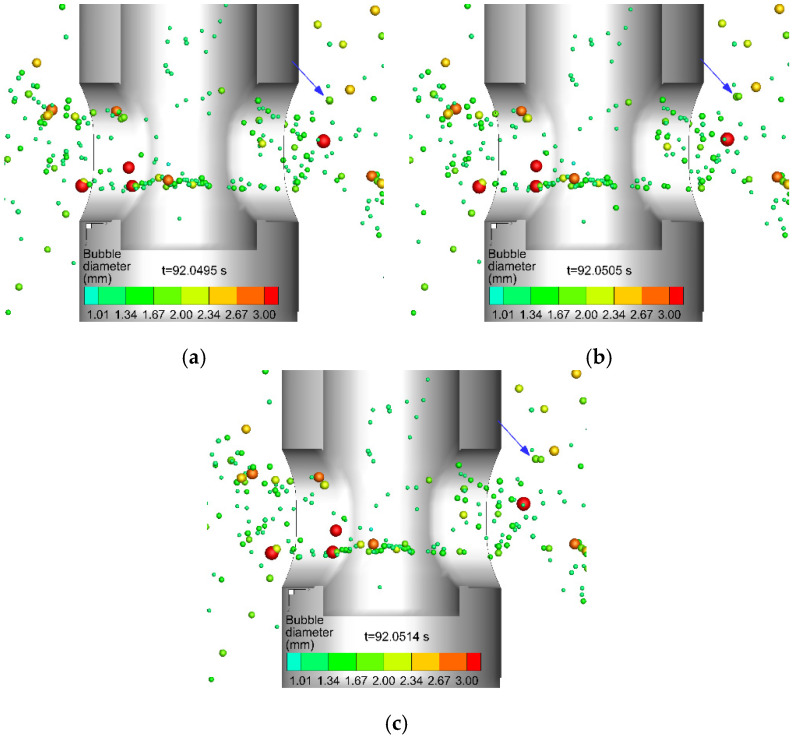
The typical process of the bounce of bubbles (marked by a blue arrow), (**a**) t = 92.0495 s, (**b**) t = 92.0505 s, and (**c**) t = 92.0514 s.

The collision of bubbles originated from the random movement of bubbles under turbulent flow. The different velocities between the bubbles make collisions between the bubbles possible. The outcome of the collision mainly included coalescence and bouncing. Figure 6 and Figure 7 show the typical process of coalescence and bouncing of bubbles near the SEN, respectively. As shown in Figure 6a, the diameter of the bubble marked by the arrow was less than 2.34 mm. The bubbles then gradually collided and grew between 2.67 and 3.0 mm in Figure 6b and larger than 3.0 mm in Figure 6c. Figure 7a,c shows the complete process of the bounce of two bubbles. The current mathematical model considering the interaction between bubbles completely reproduced the movement of bubbles in molten steel and the phenomenon of bubble coalescence and bounce during the CC process.

**Figure 8:**
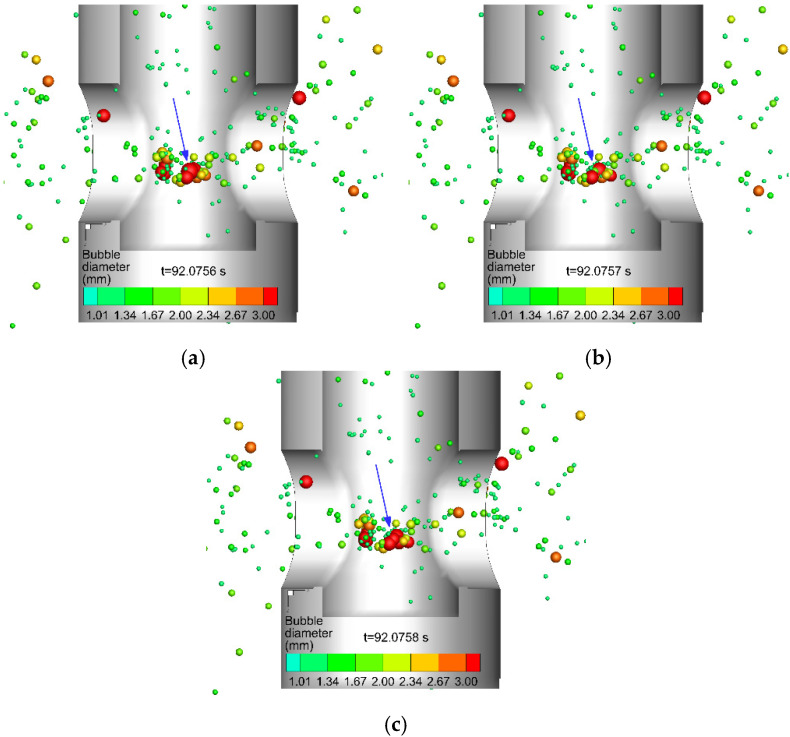
The typical process of the breakup of bubbles (marked by a blue arrow), (**a**) t = 92.0756 s, (**b**) t = 92.0757 s, and (**c**) t = 92.0758 s.

Figure 8 shows the typical process of the breakup of bubbles near the lower part of the SEN. The large bubble marked in Figure 8a was broken into small size bubbles in Figure 8b,c with the action of the fluid. In addition, some bubbles collided and grew larger. Breakup occurred when the diameter of the bubbles after coalescence exceeded the maximum diameter of the bubbles that could stably exist under the current turbulent energy dissipation rate. The strong turbulent motion of the molten steel after hitting the bottom of the SEN made the bubble breakup mainly occur near the bottom of the SEN. The breakup of bubbles was more likely to occur in the SEN region. The bubbles in the mold tended to collide.

**Figure 9:**
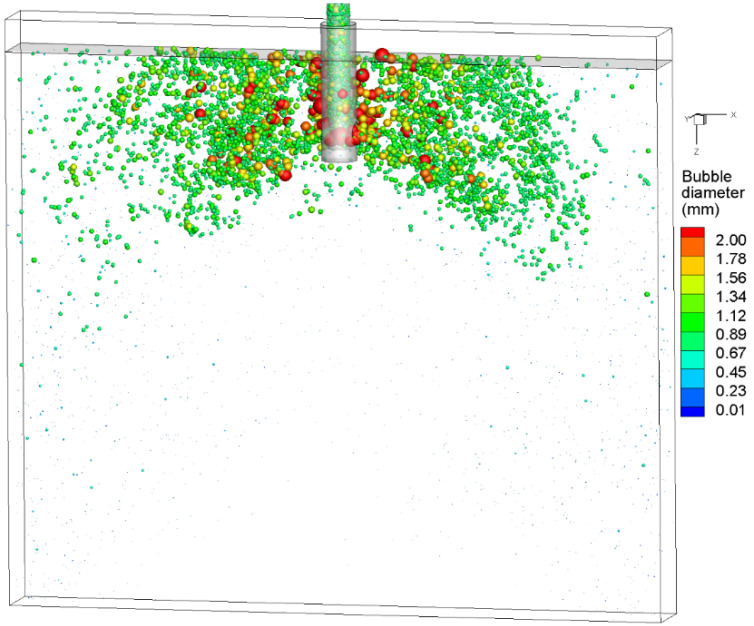
Spatial distribution of bubbles at t = 90 s.

Figure 9 shows the instantaneous spatial distribution of bubbles in the mold at t = 90 s. After the bubbles moved out of the SEN, the larger bubbles floated up near the SEN due to their greater buoyancy, while the small bubbles were distributed discretely in the entire mold with the action of the molten steel jet. Most of the bubbles floated up to the meniscus before reaching the narrow face, and overall, the bubbles were distributed in a fan shape. The distribution of bubbles in the mold was transient, including the bubble size, location, and number density. The bubbles collided and collapsed from an initial 0.71 mm into a multi-size distribution. The largest diameter exceeded 2 mm. The instantaneous spatial distribution of bubbles illustrated that the coalescence, bounce, and breakup of bubbles should be considered when predicting the two-phase flow and bubble distribution in the mold.

**Figure 10:**
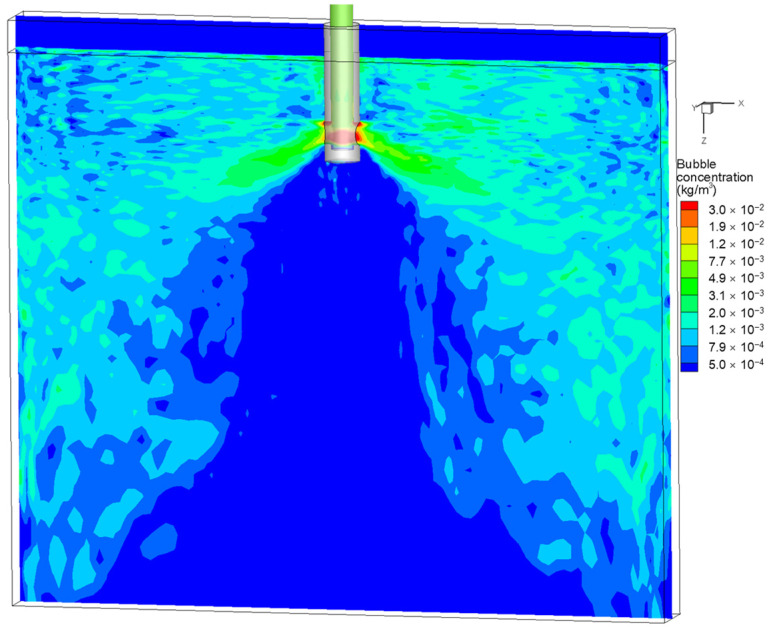
Distribution of the average bubble concentration in the mold.

Figure 10 shows the distribution of the average bubble concentration in the mold. The greatest average concentration of bubbles was in the lower part of the SEN and the upper edge of the SEN outlet. The drag force acting on the bubbles induced a relatively high bubble concentration in the jet region. In addition, the buoyancy of the larger bubbles increased the bubble concentration near the SEN. The large bubbles were broken up with the shearing of the molten steel to produce small bubbles distributed near the deep part of the mold. These small bubbles brought into the deep part of the mold were easily captured by the solidified shell to form defects.

## 5. Conclusions

In the current study, a three-dimensional mathematical model coupled with the LES turbulent model, the VOF multiphase model, and the DPM was developed to investigate the transient two-phase and spatial distribution of bubbles in a CC mold. The following conclusions were obtained:

1. The current mathematical model successfully predicted the transient asymmetric two-phase flow in the mold. The flow pattern in the mold was a typical double-roll flow with a 0.425 m/min casting speed and a 90 mL/min gas flow rate. The meniscus speed increased first and then decreased from the SEN to the narrow face, and the maximum speed appeared near the 1/4 width of the mold.
2. The interaction between the liquid and bubbles, and the coalescence, bounce, and breakup of bubbles, should be included to achieve an accurate prediction of the spatial distribution of bubbles.
3. Due to the violent movement of the fluid in the SEN, most of the breakup of the bubbles occurred in the SEN region. At the same time, the probability of the coalescence and bounce of bubbles in the SEN also increased. The bubbles in the mold tended to collide.
4. The bubbles collided and collapsed from an initial 0.71 mm into a multi-size distribution. The average bubble diameter was about 0.6 mm near the nozzle and gradually decreased to 0.05 mm from the nozzle to the narrow face. Most of the bubbles floated up to the meniscus before reaching the narrow face, and overall, the bubbles were distributed in a fan shape. The greatest average concentration of bubbles was in the lower part of the SEN and the upper edge of the SEN outlet.

## Data Availability

Not applicable.
